# What Can Parasites Tell Us About the Pathogenesis and Treatment of Asthma and Allergic Diseases

**DOI:** 10.3389/fimmu.2020.02106

**Published:** 2020-09-11

**Authors:** Sina Bohnacker, Fabiana Troisi, Marta de los Reyes Jiménez, Julia Esser-von Bieren

**Affiliations:** Center of Allergy and Environment (ZAUM), Technical University of Munich and Helmholtz Center Munich, Munich, Germany

**Keywords:** helminths, inflammation, macrophage, asthma, immune regulation, allergy, helminth molecules, type 2 immunity

## Abstract

The same mechanisms that enable host defense against helminths also drive allergic inflammation. This suggests that pathomechanisms of allergic diseases represent evolutionary old responses against helminth parasites and that studying antihelminth immunity may provide insights into pathomechanisms of asthma. However, helminths have developed an intricate array of immunoregulatory mechanisms to modulate type 2 immune mechanisms. This has led to the hypothesis that the lack of helminth infection may contribute to the rise in allergic sensitization in modern societies. Indeed, the anti-inflammatory potential of helminth (worm) parasites and their products in allergy and asthma has been recognized for decades. As helminth infections bring about multiple undesired effects including an increased susceptibility to other infections, intended helminth infection is not a feasible approach to broadly prevent or treat allergic asthma. Thus, the development of new helminth-based biopharmaceutics may represent a safer approach of harnessing type 2–suppressive effects of helminths. However, progress regarding the mechanisms and molecules that are employed by helminths to modulate allergic inflammation has been relatively recent. The scavenging of alarmins and the modulation of lipid mediator pathways and macrophage function by helminth proteins have been identified as important immunoregulatory mechanisms targeting innate immunity in asthma and allergy. In addition, by regulating the activation of dendritic cells and by promoting regulatory T-cell responses, helminth proteins can counterregulate the adaptive T helper 2 cell response that drives allergic inflammation. Despite these insights, important open questions remain to be addressed before helminth molecules can be used for the prevention and treatment of asthma and other allergic diseases.

## Introduction

Helminth infections affect about 2 billion people worldwide, and children in developing countries are particularly susceptible (1). Depending on parasite burden, helminth infections can be asymptomatic or induce pathology in the host, with malnutrition, anemia, educational loss, and cognitive deficits as major consequences (2–4).

Helminths usually infest their host as tissue-migratory larvae, which establish niches in the lung, skin, liver, or intestine, where they develop, mate, and release new infectious offspring. The host plays a critical role in this life cycle and represents a vehicle for the spread of the parasite. During evolution, helminths have learned to suppress host defense and establish chronic infections that can endure up to 20 years (5). Helminths typically induce a host protective type 2 cell–mediated immunity, which limits type 1 inflammation, reduces host tissue damage, and ensures parasite survival (6). Helminth-induced type 2 immune responses are initiated by the damaged epithelium, which secretes alarmins [interleukin 25 (IL-25), IL-33, and thymic stromal lymphopoietin] that activate and recruit type 2 innate lymphoid cells (ILCs2) and CD4^+^ T helper 2 (T_H_2) lymphocytes. The production of type 2 cytokines (IL-4, IL-5, IL-10, and IL-13), as well as granulocyte-macrophage colony-stimulating factor (GM-CSF), by these cells induces eosinophilia, M2 macrophage polarization, and the secretion of immunoglobulin G1 (IgG1), IgG4, and IgE (7–11).

A type 2 immune response is also a hallmark of asthma and allergy, suggesting that host defense and repair mechanisms of antihelminth immunity have implications for the pathogenesis and treatment of these inflammatory diseases. Epidemiological evidence on the reciprocity between helminthiases and chronic inflammatory diseases has implicated helminth infections in the prevention of allergy and asthma [see previous reviews (12–14)]. Helminths produce molecules with powerful immunomodulatory activities such as the anti-inflammatory protein-2 (AIP-2) in hookworms, the transforming growth factor β (TGF-β) mimic (Hp-TGM), the alarmin release inhibitor (Hp-ARI), or the enzyme glutamate dehydrogenase (Hpb-GDH) in the nematode *Heligmosomoides polygyrus* (15–18). The anti-inflammatory effects of helminth products observed in experimental models of asthma prompt a better investigation of helminth-(product)–driven regulation of type 2 inflammation and its underlying mechanisms of action in human settings. Current research aims to translate promising findings from rodent models to human disease and to ultimately develop helminth-based biotherapeutics for the prevention and therapy of allergy and asthma.

## Epidemiological Evidence for Protective Roles of Helminths in Allergy and Asthma

Helminths exert diverse effects on asthma and allergies depending on the species, parasite load, and time of infection (19, 20). Some parasites trigger or worsen asthma and allergic symptoms, whereas others tend to reduce the risk of these diseases (21).

*Ascaris lumbricoides* is a gastrointestinal parasite that passages through the lung. Studies in several countries have shown an association between *Ascaris* infection, asthma, and aeroallergen sensitization (22–24), which also correlated with *Ascaris*-specific IgE (sIgE) (25–27). A high prevalence of asthma and wheezing was particularly observed among *Ascaris*-infected children (28, 29). Similarly, infection with *Strongyloides* and *Toxocara* species correlates positively with allergic airway disorders. Infection with the intestinal parasite *Strongyloides stercoralis* was associated with an increased risk of asthma and its exacerbation (21, 30, 31) and *Toxocara* species infection resulted in increased allergy and asthma prevalence in children, which positively correlated with serum IgE levels (32–34). Thus, some helminth species trigger mechanisms such as the production of cross-reactive IgE or inflammatory mediators that promote allergic sensitization and/or asthma symptoms. A detailed understanding of how parasites drive allergic inflammation may provide important insights into pathomechanisms and therapeutic targets of allergy and asthma.

However, other epidemiological studies have shown a lower prevalence of asthma and allergic disorders during chronic intestinal helminth infections (35–37). Hookworm infection appears to be particularly protective (21), whereas the results for other parasites vary, depending on study design and the assessed outcomes. In several studies, deworming of chronically infected people increased allergic reactions and overall responsiveness of patients’ immune cells (38–41), and long-term antihelminthic treatment increased skin prick test reactivity to mite in *Ascaris* species and *Trichuris* species*–*infected children, as well as in allergic rhinitis patients (38–40). However, effects on asthma or rhinitis symptoms were not assessed in these studies. Direct evidence for helminth-driven modulation of allergic diseases in humans came from a multitude of studies on *Schistosoma* species infection. Children infected with *Schistosoma haematobium* displayed reduced skin prick test reactivity to house dust mite (HDM) and other aeroallergens (42) and lower allergic responses to mite were observed in *Schistosoma mansoni–*infected individuals (43). Allergy-protective effects of helminths were related to the intensity and chronicity of the infection, as well as parasite burden (36, 44, 45). Furthermore, in the presence of *S. mansoni*, peripheral blood mononuclear cells from asthmatic patients released a lower amount of inflammatory type 2 cytokines and higher levels of anti-inflammatory IL-10 (46). A lower hospitalization rate was observed for asthmatic patients infected with *S. mansoni*, suggesting that infection may reduce asthma morbidity (47).

In summary, the detrimental or protective effects of helminthiases on asthma and allergy depend on the parasite species, the duration of the infection, and the immunological context. These diverse effects may be due to different antigen or mediator repertoires, which affect hallmark type 2 responses such as eosinophil recruitment, the activation of allergen-specific T_H_2 cells, or IgE class switching. Worm molecules may also exert a different propensity for uptake by antigen-presenting cells and thus differentially regulate the induction of T cell responses. Finally, environmental factors, the presence of coinfections, and microbiota composition influence the immune response toward helminth parasites, resulting in different outcomes in helminth-infected individuals from different locations (48–50).

## Immunomodulation of Asthma and Allergic Diseases by Helminth Molecules

As helminth infection has been implicated in the prevention of allergy and asthma, experimental infection with helminths has been used in humans and animals to test potential therapeutic effects. Although rodent studies have demonstrated that helminth infection ameliorates allergic inflammation, clinical trials have not found the same benefits (51–54). Encouraging results regarding the modulation of the immune response during asthma were observed in experimental infections with *Schistosoma* species, *H. polygyrus*, and *Nippostrongylus brasiliensis. S. mansoni* and *Schistosoma japonicum* are natural human parasites that showed anti-inflammatory effects in models of ovalbumin (OVA) and HDM allergy (45, 55–57). Protection against allergic airway inflammation (AAI) in *Schistosoma*-infected mice was associated with the upregulation of IL-10, downregulation of IL-5, and induction of regulatory T cells (Tregs), which together induce a modified type 2 immune response (58–60). Induction of Tregs and IL-10 production is also implicated in allergy-suppressive actions of the gastrointestinal mouse parasite *H. polygyrus* (61–64). Infection with *H. polygyrus* suppressed airway inflammation, by reducing eosinophil recruitment, and this effect was associated with Treg and Breg expansion and the upregulation of anti-inflammatory IL-10 (63, 65). IL-10–dependent prevention of allergy has also been observed with the parasite *N. brasiliensis*, in a model of OVA-induced airway hyperresponsiveness in rats. These studies suggest shared allergy-suppressive mechanisms among different parasite species (66).

**FIGURE 1:**
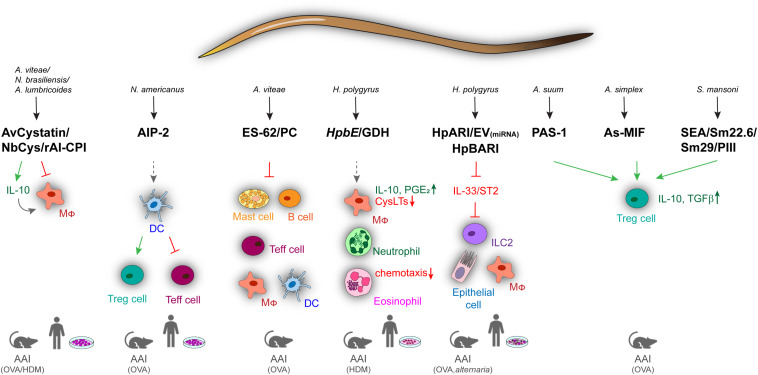
Overview of immune regulatory helminth molecules and their mechanisms of action in mouse models of allergic airway inflammation and in human *in vitro* models. Immunomodulators from different helminths can act on a variety of cells ranging from innate to adaptive and effector immune cells. Blocking of signaling is shown by red arrows, induction by green, and modulation by spaced, gray arrows. AAI, allergic airway inflammation; AIP-2, anti-inflammatory protein 2; As, *A. simplex*; Av, *A. vitae*; Cys, cystatin; DC, dendritic cell; Ev, Extracellular vesicles; GDH, glutamate dehydrogenase; HDM, house dust mite; HpARI, *H. polygyrus* Alarmin Release Inhibitor; HpBARI, *H. polygyrus* Binds Alarmin Receptor and Inhibits; *HpbE*, *H. polygyrus* extract; Mφ, macrophage; MIF, macrophage migration inhibitory factor; Nb, *N. brasiliensis*; OVA, ovalbumin; PC, phosphocholine; SEA, schistosome soluble egg antigen; Sm, *S. mansoni*.

Although animal models of helminth infection have contributed to the understanding of parasite-driven immune regulation in asthma and allergy, deeper insights into immunomodulatory effects of helminths have been provided by studying active molecules produced by parasites. The systematic analysis of parasite products by the help of proteomics and genomics has identified a comprehensive collection of helminth-derived molecules with immunomodulatory effects on asthma and allergic diseases (Figure 1). One of the best characterized helminth-derived immunomodulators is ES-62, a phosphorylcholine (PC)–containing glycoprotein secreted by the parasitic filarial nematode *Acanthocheilonema viteae*. ES-62 has shown protective effects in mouse models of asthma, lung fibrosis, and rheumatoid arthritis (67–70), with its immunomodulatory capacity depending on the PC moiety (71). Through PC modification, ES-62 can act on a variety of cells of the immune system, ranging from mast cells (MCs), macrophages, dendritic cells (DCs) to B cells, to affect intracellular pathways associated with antigen receptor and TLR signaling (67, 72–75). In MCs, ES-62 inhibits high-affinity IgE receptor (FcεRI)–induced degranulation, resulting in reduced ear swelling and hypersensitivity in a mouse model of oxazolone-induced skin inflammation. The suppression of MC activity by ES-62 further diminished airway hyperresponsiveness, lung pathology, and eosinophilia during OVA-induced AAI (67). The regulatory effects of ES-62 were mediated by the suppression of OVA-specific CD4^+^ T cell proliferation, concomitant with decreased production of IL-4, IL-13, and interferon γ (IFN-γ) (76). The regulatory potential of ES-62 on MCs depended on the inhibition of MyD88-mediated signaling downstream of TLR4 and FcεRI3, which was partially dependent on IL-33/ST2 signaling (75, 77). The suppression of IL-33 signaling was also described as a key mechanism underlying the *H. polygyrus*–driven modulation of type 2 immune responses. This effect is mediated by the secretion of an Alarmin Release Inhibitor (HpARI), which binds and blocks IL-33, and by the recently discovered Binds Alarmin Receptor and Inhibits (HpBARI) protein, which blocks the IL-33 ST2 receptor in mice and human cells (18, 78). HpARI was shown to hamper IL-33 release in human lung explants and in a human IL-33 transgenic mouse model after *Alternaria* allergen administration (18), whereas HpBARI inhibited eosinophil recruitment after *Alternaria* allergen administration (78). Another undefined *H. polygyrus* product was able to downregulate IL-33 production through the induction of IL-1β, thus promoting parasite chronicity (79). In *Alternaria*-induced AAI, *H. polygyrus* downregulated the IL-33 receptor via releasing extracellular vesicles containing microRNAs, resulting in reduced eosinophilia and improved lung function (18, 80, 81). These results indicate that vesicle release represents an efficient way to deliver immunomodulatory molecules to host immune cells. Similar to scavenging of IL-33 by HpARI, the recently identified protein p43 from *Trichuris muris* can bind IL-13 and thereby inhibit parasite expulsion (82), raising the question if this molecule can also modulate IL-13–driven airway inflammation.

Another conserved mechanism of helminth-driven immune regulation is the use of cysteine protease inhibitors (cystatins). Mammalian cysteine proteases are required for proteolytic processing of antigens, enabling presentation on MHC class II molecules and effective T cell responses. Cystatins from *A. viteae*, *Brugia malayi*, *N. brasiliensis*, *Onchocerca volvulus*, *Clonorchis sinensis*, *A. lumbricoides*, *H. polygyrus*, and *Litomosoides sigmodontis* have been shown to interfere with this process to evade antigen-induced immunity (83–94). AvCystatin from *A. viteae* mitigated airway inflammation and colitis in mice through the induction of IL-10–producing macrophages (93) and reduced pollen-specific responses in lymphocytes from allergic patients (94). Cystatin from *N. brasiliensis* (NbCys) dampened OVA-specific splenocyte proliferation, as well as IgE and cytokine production by inhibiting cathepsins L and B (89). Similar effects were observed for cystatin (rAl-CPI) from *A. lumbricoides*, which decreased T_H_2 cytokine and IgE production in a mouse model of HDM-induced AAI (92).

A large repertoire of immunomodulatory molecules is also present in the egg stage of some parasites. Schistosome soluble egg antigen (SEA) from *S. japonicum* showed inhibitory effects on the development of airway inflammation in a CD4^+^ CD25^+^ T cell–dependent manner during OVA-induced asthma in mice (95). In the same model, antigens from *S. mansoni* (Sm22.6, Sm29, and PIII) reduced airway inflammation, eosinophilia, OVA-specific IgE levels, and T_H_2 cytokine production in the BAL. The beneficial effects of Sm22.6 were due to the induction of IL-10, similar to the *S. mansoni* egg glycoprotein IPSE/α-1, which induced IL-10–producing Bregs (96). In contrast, SM22.6 and PIII triggered the expansion of CD4^+^Foxp3^+^ T cells suggesting that both Treg and Breg cells are involved in the modulation of type 2 inflammation by SEA (97).

Helminth molecules can also mimic host-derived mediators. *H. polygyrus* or administration of its excretory–secretory products (HES) induces Treg cells, suppressing effector cell proliferation *in vitro* and AAI *in vivo*. This regulatory response was mediated by Hp-TGM, a protein with TGF-β–like activity (15, 64). TGH-2 from *B. malayi* similarly activated TGF-β pathways, suggesting TGF-β signaling as a shared immunomodulatory mechanism among parasite species (98). *B. malayi*, *Ancylostoma ceylanicum*, *Trichinella spiralis*, and *Anisakis simplex* also produce homologs of the mammalian cytokine macrophage migration inhibitory factor (MIF) (99–103). MIF homologs from *B. malayi* (99) and *T. spiralis* (100) functionally reflect host MIF proteins, e.g., regarding chemotactic effects on monocytes, whereas the MIF homolog from *A. simplex* (As-MIF) showed direct anti-inflammatory activity on OVA-induced AAI, where it suppressed the production of T_H_2 cytokines (IL-4, IL-5, and IL-13), as well as eosinophilia and goblet cell hyperplasia in the airways. These effects were again associated with the recruitment of CD4^+^CD25^+^Foxp3^+^ T cells and the upregulation of IL-10 and TGF-β (102, 103).

Treg cell induction *in vivo* was also observed for an excretory/secretory protein of *Ascaris suum* (PAS-1), which inhibited airway inflammation in a murine model of OVA-induced AAI by decreasing eosinophilia and T_H_2 cytokines in the BAL, as well as OVA-specific serum IgE (104). PAS-1 also abrogated airway inflammation and airway hyperreactivity induced by the proinflammatory *A. suum* molecule APAS-3 by reducing the production of proinflammatory cytokines in the airways and IgG1 and IgE levels in the serum (105). The amelioration of OVA-induced asthma by PAS-1 was mediated by IL-10/TGF-β–producing Treg cells (CD4^+^CD25^+^) and IFN-γ–producing CD8^+^ T cells (104, 106). Thus, many helminth molecules target IL-10, TGF-β, and IFN-γ, which efficiently suppress type 2 cytokine and antibody responses involved in antihelminth immunity and allergic inflammation (107).

Recently, a metalloprotease (TIMP)–like protein from *Necator americanus* (AIP-2) with Treg-mediated anti-inflammatory effects on AAI was identified. AIP-2 did not suppress matrix metalloprotease catalytic activity, but modulated the activity of CD103^+^ DCs that reduced the expression of costimulatory markers and expanded Treg cells. Thus, administration of AIP-2 reduced eosinophil recruitment, type 2 cytokine (IL-5, IL-13) production in the airways, and OVA-specific IgE in the serum. Importantly, AIP-2 also inhibited the proliferation of T effector cells from the blood of human HDM allergic patients (17).

Another recent study showed that in addition to products of the adult L5 stage of *H. polygyrus* (e.g., HES, HpARI), a preparation of the infective larval (L3) stage could protect mice against the development of AAI. The *H. polygyrus* larval extract (*HpbE*) and its active protein component, *Hpb* GDH, efficiently suppressed HDM-induced AAI *in vivo*. In particular, *HpbE* and recombinant *Hpb* GDH modulated the arachidonic acid metabolism of macrophages, inducing an anti-inflammatory, type 2 suppressive eicosanoid profile (16). *HpbE*-/GDH-treated macrophages exhibited high IL-10 and prostaglandin E_2_ (PGE_2_) production, but low production of proinflammatory leukotrienes, which are key mediators of AAI (16, 108). Macrophage-derived PGE_2_ was particularly important for the *HpbE*-driven regulation of AAI in this study, and another study found that also helminths themselves can produce this immunomodulatory mediator (109). The *HpbE*-induced eicosanoid switch was largely mediated through nuclear factor κB, p38 mitogen-activated protein kinase, hypoxia-inducible factor 1α, and the cyclooxygenase-2 pathway. Finally, *HpbE* reduced the chemotaxis of granulocytes from patients suffering from type 2 airway inflammation (16).

Together, these studies reveal that helminth molecules are efficient modulators of the innate and adaptive immune responses that drive AAI.

## Discussion

Helminths have unique immune regulatory potential, and understanding the complex array of immune responses triggered by these parasites may be instrumental for the diagnosis, prevention, and treatment of type 2 inflammatory diseases, such as allergic asthma. Identifying the molecules and mechanisms that determine whether a parasite will promote or suppress allergic inflammation may foster both the definition and targeting of pathomechanisms of chronic type 2 inflammation. Parasitic infections influence immunity and inflammation by a variety of molecular and cellular mechanisms, including the induction of Treg cells and regulatory macrophages, producing anti-inflammatory mediators, such as TGF-β, IL-10, and PGE_2_, with beneficial effects in experimental models of asthma. However, the translation of these results from rodents to humans is not trivial. For instance, little is known about the correct dose or duration of parasite infection required for protective effects in humans. Safety concerns about detrimental effects of parasite infection limit clinical trials, and high immunological variation, e.g., due to different genetic background, complicates the interpretation of data from experimental helminth infection in humans. Indeed, not all studies show an impact of helminth infection or deworming on allergic inflammation (110, 111), which is in line with the lack of a therapeutic effect of intended helminth infection on AAI in humans (51–53) [for a comprehensive review, see Evans and Mitre (112)]. It is important to note that epidemiological studies commonly assess effects of helminth infection on skin prick test reactivity (e.g., atopy) rather than asthma symptoms, which may explain disparities between different studies.

Safety concerns regarding live helminth infections may be overcome by the identification and characterization of helminth-derived anti-inflammatory molecules, which may be developed as biotherapeutics. Therapeutic approaches exploiting the immunomodulatory potential of helminths, while avoiding infection-related side effects, represent an attractive treatment option for major chronic airway diseases. The identification of the cellular and molecular pathways targeted by helminth molecules (e.g., T cells, DCs, TLR-/IL-33 signaling) should aid the discovery of new worm-based drugs. Such drugs will have to be delivered preferentially locally, i.e., to the inflamed tissue at an optimal dose, route, and frequency of administration, which remains to be determined for each molecule. The recent identification of immune regulatory molecules that reduce AAI upon local delivery and simultaneously act on key human cells involved in asthma (e.g., epithelial cells, macrophages, eosinophils) (16–18) justifies the hope that effective topical helminth-based biotherapeutics can be developed. Formulation for local delivery into the airways represents a vital alternative to current biologics or oral corticosteroids that today represent the standard treatment for more severe forms of type 2 airway inflammation. However, before helminth-derived molecules can reach the clinics, there are several hurdles to be cleared. This particularly includes the immunogenicity of helminth molecules, potential proinflammatory side effects, as well as their half-life in the human organism. Reducing the immunogenicity of foreign helminth molecules represents a major challenge that may, e.g., be tackled by packaging immune regulatory proteins into nanocarriers for targeted delivery to a specific cell type or by designing non-immunogenic (humanized) mutants. Despite these challenges, significant scientific progress has been made to turn worm molecules into drug candidates. The unique and diverse modes of action of helminth-derived molecules make them promising candidates to become the next generation of biotherapeutics for the treatment of type 2 inflammatory disorders.

## Author Contributions

SB, FT, MR, and JE wrote the manuscript, SB and FT prepared the figures. All authors contributed to the article and approved the submitted version.

## Conflict of Interest

The authors declare that the research was conducted in the absence of any commercial or financial relationships that could be construed as a potential conflict of interest.

## References

[1] Hotez PJ Brindley PJ Bethony JM King CH Pearce EJ Jacobson J. Helminth infections: the great neglected tropical diseases. *J Clin Invest.* (2008) 118:1311–21. 10.1172/JCI34261 18382743 PMC2276811

[2] Osazuwa F Ayo OM Imade P. A significant association between intestinal helminth infection and anaemia burden in children in rural communities of Edo state, Nigeria. *N Am J Med Sci.* (2011) 3:30–4. 10.4297/najms.2011.330 22540060 PMC3336930

[3] Pabalan N Singian E Tabangay L Jarjanazi H Boivin MJ Ezeamama AE. Soil-transmitted helminth infection, loss of education and cognitive impairment in school-aged children: a systematic review and meta-analysis. *PLoS Negl Trop Dis.* (2018) 12:e0005523. 10.1371/journal.pntd.0005523 29329288 PMC5766095

[4] Stephenson LS Latham MC Ottesen EA. Malnutrition and parasitic helminth infections. *Parasitology.* (2000) 121:S23–38. 10.1017/S0031182000006491 11386688

[5] Maizels RM McSorley HJ. Regulation of the host immune system by helminth parasites. *J Allergy Clin Immunol.* (2016) 138:666–75. 10.1016/j.jaci.2016.07.007 27476889 PMC5010150

[6] Maizels RM Yazdanbakhsh M. Immune regulation by helminth parasites: cellular and molecular mechanisms. *Nat Rev Immunol.* (2003) 3:733–44. 10.1038/nri1183 12949497

[7] Anthony RM Urban JF Alem F Hamed HA Rozo CT Boucher J-L Memory TH2 cells induce alternatively activated macrophages to mediate protection against nematode parasites. *Nat Med.* (2006) 12:955–60. 10.1038/nm1451 16892038 PMC1955764

[8] Esser-von Bieren J Mosconi I Guiet R Piersgilli A Volpe B Chen F Antibodies trap tissue migrating helminth larvae and prevent tissue damage by driving IL-4Rα-independent alternative differentiation of macrophages. *PLoS Pathog.* (2013) 9:e1003771. 10.1371/journal.ppat.1003771 24244174 PMC3828184

[9] Fallon PG Jolin HE Smith P Emson CL Townsend MJ Fallon R IL-4 induces characteristic Th2 responses even in the combined absence of IL-5, IL-9, and IL-13. *Immunity.* (2002) 17:7–17. 10.1016/s1074-7613(02)00332-1 12150887

[10] Gounni AS Lamkhioued B Ochiai K Tanaka Y Delaporte E Capron A High-affinity IgE receptor on eosinophils is involved in defence against parasites. *Nature.* (1994) 367:183–6. 10.1038/367183a0 8114916

[11] Voehringer D Reese TA Huang X Shinkai K Locksley RM. Type 2 immunity is controlled by IL-4/IL-13 expression in hematopoietic non-eosinophil cells of the innate immune system. *J Exp Med.* (2006) 203:1435–46. 10.1084/jem.20052448 16702603 PMC2118302

[12] Maizels RM. Parasitic helminth infections and the control of human allergic and autoimmune disorders. *Clin Microbiol Infect.* (2016) 22:481–6. 10.1016/j.cmi.2016.04.024 27172808

[13] Smallwood TB Giacomin PR Loukas A Mulvenna JP Clark RJ Miles JJ. Helminth immunomodulation in autoimmune disease. *Front Immunol.* (2017) 8:453. 10.3389/fimmu.2017.00453 28484453 PMC5401880

[14] Daniłowicz-Luebert E O’Regan NL Steinfelder S Hartmann S. Modulation of specific and allergy-related immune responses by helminths. *J Biomed Biotechnol.* (2011) 2011:1–18. 10.1155/2011/821578 22219659 PMC3248237

[15] Johnston CJC Smyth DJ Kodali RB White MPJ Harcus Y Filbey KJ A structurally distinct TGF-β mimic from an intestinal helminth parasite potently induces regulatory T cells. *Nat Commun.* (2017) 8:1741. 10.1038/s41467-017-01886-6 29170498 PMC5701006

[16] de los Reyes Jiménez M Lechner A Alessandrini F Bohnacker S Schindela S Trompette A An anti-inflammatory eicosanoid switch mediates the suppression of type-2 inflammation by helminth larval products. *Sci Transl Med.* (2020) 12:eaay0605. 10.1126/scitranslmed.aay0605 32321863

[17] Navarro S Pickering DA Ferreira IB Jones L Ryan S Troy S Hookworm recombinant protein promotes regulatory T cell responses that suppress experimental asthma. *Sci Transl Med.* (2016) 8:362ra143. 10.1126/scitranslmed.aaf8807 27797959

[18] Osbourn M Soares DC Vacca F Cohen ES Scott IC Gregory WF HpARI protein secreted by a helminth parasite suppresses interleukin-33. *Immunity.* (2017) 47:739–51.e5. 10.1016/j.immuni.2017.09.015 29045903 PMC5655542

[19] Fernandes JS Cardoso LS Pitrez PM Cruz ÁA. Helminths and asthma: risk and protection. *Immunol Allergy Clin North Am.* (2019) 39:417–27. 10.1016/j.iac.2019.03.009 31284930

[20] Maizels RM. Regulation of immunity and allergy by helminth parasites. *Allergy.* (2020) 75:524–34. 10.1111/all.13944 31187881

[21] Leonardi-Bee J Pritchard D Britton J. Asthma and current intestinal parasite infection: systematic review and meta-analysis. *Am J Respir Crit Care Med.* (2006) 174:514–23. 10.1164/rccm.200603-331OC 16778161

[22] da Silva ER Sly PD de Pereira MU Pinto LA Jones MH Pitrez PM Intestinal helminth infestation is associated with increased bronchial responsiveness in children. *Pediatr Pulmonol.* (2008) 43:662–5. 10.1002/ppul.20833 18484663

[23] Pereira MU Sly PD Pitrez PM Jones MH Escouto D Dias ACO Nonatopic asthma is associated with helminth infections and bronchiolitis in poor children. *Eur Respir J.* (2007) 29:1154–60. 10.1183/09031936.00127606 17331964

[24] Palmer LJ Celedón JC Weiss ST Wang B Fang Z Xu X. *Ascaris lumbricoides* infection is associated with increased risk of childhood asthma and atopy in rural China. *Am J Respir Crit Care Med.* (2002) 165:1489–93. 10.1164/rccm.2107020 12045121

[25] Obihara CC Beyers N Gie RP Hoekstra MO Fincham JE Marais BJ Respiratory atopic disease, *Ascaris*-immunoglobulin E and tuberculin testing in urban South African children. *Clin Exp Allergy.* (2006) 36:640–8. 10.1111/j.1365-2222.2006.02479.x 16650050

[26] Joubert JR van Schalkwyk DJ Turner KJ. *Ascaris lumbricoides* and the human immunogenic response: enhanced IgE-mediated reactivity to common inhaled allergens. *S Afr Med J.* (1980) 57:409–12. 6157196

[27] Dold S Heinrich J Wichmann HE Wjst M. *Ascaris*-specific IgE and allergic sensitization in a cohort of school children in the former East Germany. *J Allergy Clin Immunol.* (1998) 102:414–20. 10.1016/s0091-6749(98)70129-0 9768582

[28] Zaman K Takeuchi H Yunus MD El Arifeen S Chowdhury HR Baqui AH Asthma in rural Bangladeshi children. *Indian J Pediatr.* (2007) 74:539–43. 10.1007/s12098-007-0104-0 17595495

[29] Hawlader MDH Ma E Noguchi E Itoh M Arifeen SE Persson LÅ *Ascaris lumbricoids* infection as a risk factor for asthma and atopy in rural Bangladeshi children. *Trop Med Health.* (2014) 42:77–85. 10.2149/tmh.2013-19 25237284 PMC4139537

[30] Altintop L Cakar B Hokelek M Bektas A Yildiz L Karaoglanoglu M. *Strongyloides stercoralis* hyperinfection in a patient with rheumatoid arthritis and bronchial asthma: a case report. *Ann Clin Microbiol Antimicrob.* (2010) 9:27. 10.1186/1476-0711-9-27 20849666 PMC2949791

[31] Dunlap NE Shin MS Polt SS Ho KJ. Strongyloidiasis manifested as asthma. *South Med J.* (1984) 77:77–8. 10.1097/00007611-198401000-00021 6229882

[32] Buijs J Borsboom G Renting M Hilgersom WJ van Wieringen JC Jansen G Relationship between allergic manifestations and Toxocara seropositivity: a cross-sectional study among elementary school children. *Eur Respir J.* (1997) 10:1467–75. 10.1183/09031936.97.10071467 9230232

[33] Ferreira MU Rubinsky-Elefant G de Castro TG Hoffmann EHE da Silva-Nunes M Cardoso MA Bottle feeding and exposure to Toxocara as risk factors for wheezing illness among under-five Amazonian children: a population-based cross-sectional study. *J Trop Pediatr.* (2007) 53:119–24. 10.1093/tropej/fml083 17210615

[34] Cooper PJ. *Toxocara canis* infection: an important and neglected environmental risk factor for asthma? *Clin Exp Allergy.* (2008) 38:551–3. 10.1111/j.1365-2222.2008.02934.x 18241245

[35] Lynch NR Lopez RI Di Prisco-Fuenmayor MC Hagel I Medouze L Viana G Allergic reactivity and socio-economic level in a tropical environment. *Clin Allergy.* (1987) 17:199–207. 10.1111/j.1365-2222.1987.tb02004.x 3608138

[36] Scrivener S Yemaneberhan H Zebenigus M Tilahun D Girma S Ali S Independent effects of intestinal parasite infection and domestic allergen exposure on risk of wheeze in Ethiopia: a nested case-control study. *Lancet.* (2001) 358:1493–9. 10.1016/S0140-6736(01)06579-5 11705561

[37] Nyan OA Walraven GE Banya WA Milligan P Van Der Sande M Ceesay SM Atopy, intestinal helminth infection and total serum IgE in rural and urban adult Gambian communities. *Clin Exp Allergy.* (2001) 31:1672–8. 10.1046/j.1365-2222.2001.00987.x 11696042

[38] Borkow G Leng Q Weisman Z Stein M Galai N Kalinkovich A Chronic immune activation associated with intestinal helminth infections results in impaired signal transduction and anergy. *J Clin Invest.* (2000) 106:1053–60. 11032865 10.1172/JCI10182 PMC314342

[39] Lynch NR Hagel I Perez M Di Prisco MC Lopez R Alvarez N. Effect of anthelmintic treatment on the allergic reactivity of children in a tropical slum. *J Allergy Clin Immunol.* (1993) 92:404–11. 10.1016/0091-6749(93)90119-z 8360391

[40] van den Biggelaar AHJ Rodrigues LC van Ree R van der Zee JS Hoeksma-Kruize YCM Souverijn JHM Long-term treatment of intestinal helminths increases mite skin-test reactivity in Gabonese schoolchildren. *J Infect Dis.* (2004) 189:892–900. 10.1086/381767 14976607

[41] Wammes LJ Hamid F Wiria AE May L Kaisar MMM Prasetyani-Gieseler MA Community deworming alleviates geohelminth-induced immune hyporesponsiveness. *Proc Natl Acad Sci USA.* (2016) 113:12526–31. 10.1073/pnas.1604570113 27791067 PMC5098677

[42] van den Biggelaar AH van Ree R Rodrigues LC Lell B Deelder AM Kremsner PG Decreased atopy in children infected with *Schistosoma haematobium*: a role for parasite-induced interleukin-10. *Lancet.* (2000) 356:1723–7. 10.1016/S0140-6736(00)03206-2 11095260

[43] Araujo MI Lopes AA Medeiros M Cruz AA Sousa-Atta L Solé D Inverse association between skin response to aeroallergens and *Schistosoma mansoni* infection. *Int Arch Allergy Immunol.* (2000) 123:145–8. 10.1159/000024433 11060486

[44] Stein M Greenberg Z Boaz M Handzel ZT Meshesha MK Bentwich Z. The role of helminth infection and environment in the development of allergy: a prospective study of newly-arrived Ethiopian immigrants in Israel. *PLoS Negl Trop Dis.* (2016) 10:e0004208. 10.1371/journal.pntd.0004208 26752538 PMC4709081

[45] Smits HH Hammad H van Nimwegen M Soullie T Willart MA Lievers E Protective effect of *Schistosoma mansoni* infection on allergic airway inflammation depends on the intensity and chronicity of infection. *J Allergy Clin Immunol.* (2007) 120:932–40. 10.1016/j.jaci.2007.06.009 17689595

[46] Araujo MIAS Hoppe B Medeiros M Alcântara L Almeida MC Schriefer A Impaired T helper 2 response to aeroallergen in helminth-infected patients with asthma. *J Infect Dis.* (2004) 190:1797–803. 10.1086/425017 15499536

[47] Ponte EV Rasella D Souza-Machado C Stelmach R Barreto ML Cruz AA. Reduced asthma morbidity in endemic areas for helminth infections: a longitudinal ecological study in Brazil. *J Asthma.* (2014) 51:1022–7. 10.3109/02770903.2014.936454 24975567

[48] Mabbott NA. The influence of parasite infections on host immunity to co-infection with other pathogens. *Front Immunol.* (2018) 9:2579. 10.3389/fimmu.2018.02579 30467504 PMC6237250

[49] Fujimura KE Lynch SV. Microbiota in allergy and asthma and the emerging relationship with the gut microbiome. *Cell Host Microbe.* (2015) 17:592–602. 10.1016/j.chom.2015.04.007 25974301 PMC4443817

[50] Alcântara-Neves NM de SG Britto G Veiga RV Figueiredo CA Fiaccone RL Effects of helminth co-infections on atopy, asthma and cytokine production in children living in a poor urban area in Latin America. *BMC Research Notes.* (2014) 7:817. 10.1186/1756-0500-7-817 25410903 PMC4289379

[51] Bager P Arnved J Rønborg S Wohlfahrt J Poulsen LK Westergaard T *Trichuris suis* ova therapy for allergic rhinitis: a randomized, double-blind, placebo-controlled clinical trial. *J Allergy Clin Immunol.* (2010) 125:123–30.e1-3. 10.1016/j.jaci.2009.08.006 19800680

[52] Feary J Venn A Brown A Hooi D Falcone FH Mortimer K Safety of hookworm infection in individuals with measurable airway responsiveness: a randomized placebo-controlled feasibility study. *Clin Exp Allergy.* (2009) 39:1060–8. 10.1111/j.1365-2222.2009.03187.x 19400893 PMC2728895

[53] Feary JR Venn AJ Mortimer K Brown AP Hooi D Falcone FH Experimental hookworm infection: a randomized placebo-controlled trial in asthma. *Clin Exp Allergy.* (2010) 40:299–306. 10.1111/j.1365-2222.2009.03433.x 20030661 PMC2814083

[54] Mortimer K Brown A Feary J Jagger C Lewis S Antoniak M Dose-ranging study for trials of therapeutic infection with *Necator americanus* in humans. *Am J Trop Med Hyg.* (2006) 75:914–20. 17123987

[55] Qiu S Fan X Yang Y Dong P Zhou W Xu Y *Schistosoma japonicum* infection downregulates house dust mite-induced allergic airway inflammation in mice. *PLoS One.* (2017) 12:e0179565. 10.1371/journal.pone.0179565 28614408 PMC5470717

[56] Mo H Lei J Jiang Z Wang C Cheng Y Li Y *Schistosoma japonicum* infection modulates the development of allergen-induced airway inflammation in mice. *Parasitol Res.* (2008) 103:1183–9. 10.1007/s00436-008-1114-1 18654798

[57] Layland LE Straubinger K Ritter M Loffredo-Verde E Garn H Sparwasser T *Schistosoma mansoni*-mediated suppression of allergic airway inflammation requires patency and Foxp3+ Treg cells. *PLoS Negl Trop Dis.* (2013) 7:e2379. 10.1371/journal.pntd.0002379 23967364 PMC3744427

[58] van der Vlugt LEPM Labuda LA Ozir-Fazalalikhan A Lievers E Gloudemans AK Liu K-Y Schistosomes induce regulatory features in human and mouse CD1d(hi) B cells: inhibition of allergic inflammation by IL-10 and regulatory T cells. *PLoS One.* (2012) 7:e30883. 10.1371/journal.pone.0030883 22347409 PMC3275567

[59] Mangan NE van Rooijen N McKenzie ANJ Fallon PG. Helminth-modified pulmonary immune response protects mice from allergen-induced airway hyperresponsiveness. *J Immunol.* (2006) 176:138–47. 10.4049/jimmunol.176.1.138 16365404

[60] Schmiedel Y Mombo-Ngoma G Labuda LA Janse JJ de Gier B Adegnika AA CD4+CD25hiFOXP3+ regulatory T cells and cytokine responses in human Schistosomiasis before and after treatment with praziquantel. *PLoS Negl Trop Dis.* (2015) 9:e0003995. 10.1371/journal.pntd.0003995 26291831 PMC4546370

[61] Hartmann S Schnoeller C Dahten A Avagyan A Rausch S Lendner M Gastrointestinal nematode infection interferes with experimental allergic airway inflammation but not atopic dermatitis. *Clin Exp Allergy.* (2009) 39:1585–96. 10.1111/j.1365-2222.2009.03290.x 19508324

[62] Kitagaki K Businga TR Racila D Elliott DE Weinstock JV Kline JN. Intestinal helminths protect in a murine model of asthma. *J Immunol.* (2006) 177:1628–35. 10.4049/jimmunol.177.3.1628 16849471

[63] Wilson MS Taylor MD Balic A Finney CAM Lamb JR Maizels RM. Suppression of allergic airway inflammation by helminth-induced regulatory T cells. *J Exp Med.* (2005) 202:1199–212. 10.1084/jem.20042572 16275759 PMC2213237

[64] Grainger JR Smith KA Hewitson JP McSorley HJ Harcus Y Filbey KJ Helminth secretions induce de novo T cell Foxp3 expression and regulatory function through the TGF-β pathway. *J Exp Med.* (2010) 207:2331–41. 10.1084/jem.20101074 20876311 PMC2964568

[65] Gao X Ren X Wang Q Yang Z Li Y Su Z Critical roles of regulatory B and T cells in helminth parasite-induced protection against allergic airway inflammation. *Clin Exp Immunol.* (2019) 198:390–402. 10.1111/cei.13362 31397879 PMC6857085

[66] Wohlleben G Trujillo C Müller J Ritze Y Grunewald S Tatsch U Helminth infection modulates the development of allergen-induced airway inflammation. *Int Immunol.* (2004) 16:585–96. 10.1093/intimm/dxh062 15039389

[67] Melendez AJ Harnett MM Pushparaj PN Wong WF Tay HK McSharry CP Inhibition of FcεRI-mediated mast cell responses by ES-62, a product of parasitic filarial nematodes. *Nat Med.* (2007) 13:1375–81. 10.1038/nm1654 17952092

[68] Rzepecka J Coates ML Saggar M Al-Riyami L Coltherd J Tay HK Small molecule analogues of the immunomodulatory parasitic helminth product ES-62 have anti-allergy properties. *Int J Parasitol.* (2014) 44:669–74. 10.1016/j.ijpara.2014.05.001 24929132 PMC4119935

[69] Suckling CJ Mukherjee S Khalaf AI Narayan A Scott FJ Khare S Synthetic analogues of the parasitic worm product ES-62 reduce disease development in in vivo models of lung fibrosis. *Acta Tropica.* (2018) 185:212–8. 10.1016/j.actatropica.2018.05.015 29802846

[70] Doonan J Lumb FE Pineda MA Tarafdar A Crowe J Khan AM Protection against arthritis by the parasitic worm product ES-62, and its drug-like small molecule analogues, is associated with inhibition of osteoclastogenesis. *Front Immunol.* (2018) 9:1016. 10.3389/fimmu.2018.01016 29867986 PMC5967578

[71] Goodridge HS McGUINESS S Houston KM Egan CA Al-Riyami L Alcocer MJC Phosphorylcholine mimics the effects of ES-62 on macrophages and dendritic cells. *Parasite Immunol.* (2007) 29:127–37. 10.1111/j.1365-3024.2006.00926.x 17266740

[72] Goodridge HS Wilson EH Harnett W Campbell CC Harnett MM Liew FY. Modulation of macrophage cytokine production by ES-62, a secreted product of the filarial nematode *Acanthocheilonema viteae*. *J Immunol.* (2001) 167:940–5. 10.4049/jimmunol.167.2.940 11441102

[73] Goodridge HS Marshall FA Else KJ Houston KM Egan C Al-Riyami L Immunomodulation via novel use of TLR4 by the filarial nematode phosphorylcholine-containing secreted product, ES-62. *J Immunol.* (2005) 174:284–93. 10.4049/jimmunol.174.1.284 15611251

[74] Marshall FA Watson KA Garside P Harnett MM Harnett W. Effect of activated antigen-specific B cells on ES-62-mediated modulation of effector function of heterologous antigen-specific T cells in vivo. *Immunology.* (2008) 123:411–25. 10.1111/j.1365-2567.2007.02706.x 17961164 PMC2433340

[75] Pineda MA Lumb F Harnett MM Harnett W. ES-62, a therapeutic anti-inflammatory agent evolved by the filarial nematode *Acanthocheilonema viteae*. *Mol Biochem Parasitol.* (2014) 194:1–8. 10.1016/j.molbiopara.2014.03.003 24671112

[76] Marshall FA Grierson AM Garside P Harnett W Harnett MM. ES-62, an immunomodulator secreted by filarial nematodes, suppresses clonal expansion and modifies effector function of heterologous antigen-specific T cells in vivo. *J Immunol.* (2005) 175:5817–26. 10.4049/jimmunol.175.9.5817 16237074

[77] Ball DH Al-Riyami L Harnett W Harnett MM. IL-33/ST2 signalling and crosstalk with FcεRI and TLR4 is targeted by the parasitic worm product, ES-62. *Sci Rep.* (2018) 8:4497. 10.1038/s41598-018-22716-9 29540770 PMC5852134

[78] Vacca F Chauché C Jamwal A Hinchy EC Heieis G Webster H A helminth-derived suppressor of ST2 blocks allergic responses. *eLife.* (2020) 9:e54017. 10.7554/eLife.54017 32420871 PMC7234810

[79] Zaiss MM Maslowski KM Mosconi I Guenat N Marsland BJ Harris NL. IL-1beta suppresses innate IL-25 and IL-33 production and maintains helminth chronicity. *PLoS Pathog.* (2013) 9:e1003531. 10.1371/journal.ppat.1003531 23935505 PMC3731249

[80] Coakley G McCaskill JL Borger JG Simbari F Robertson E Millar M Extracellular vesicles from a helminth parasite suppress macrophage activation and constitute an effective vaccine for protective immunity. *Cell Rep.* (2017) 19:1545–57. 10.1016/j.celrep.2017.05.001 28538175 PMC5457486

[81] Buck AH Coakley G Simbari F McSorley HJ Quintana JF Le Bihan T Exosomes secreted by nematode parasites transfer small RNAs to mammalian cells and modulate innate immunity. *Nat Commun.* (2014) 5:5488. 10.1038/ncomms6488 25421927 PMC4263141

[82] Bancroft AJ Levy CW Jowitt TA Hayes KS Thompson S Mckenzie EA The major secreted protein of the whipworm parasite tethers to matrix and inhibits interleukin-13 function. *Nat Commun.* (2019) 10:2344. 10.1038/s41467-019-09996-z 31138806 PMC6538607

[83] Coronado S Barrios L Zakzuk J Regino R Ahumada V Franco L A recombinant cystatin from *Ascaris lumbricoides* attenuates inflammation of DSS-induced colitis. *Parasite Immunol.* (2017) 39:e12425. 10.1111/pim.12425 28295446

[84] Jang SW Cho MK Park MK Kang SA Na B-K Ahn SC Parasitic helminth cystatin inhibits DSS-induced intestinal inflammation via IL-10 ^+^ F4/80 ^+^ macrophage recruitment. *Korean J Parasitol.* (2011) 49:245. 10.3347/kjp.2011.49.3.245 22072824 PMC3210841

[85] Wang S Xie Y Yang X Wang X Yan K Zhong Z Therapeutic potential of recombinant cystatin from *Schistosoma japonicum* in TNBS-induced experimental colitis of mice. *Parasites Vectors.* (2016) 9:6. 10.1186/s13071-015-1288-1 26728323 PMC4700642

[86] Manoury B Gregory WF Maizels RM Watts C. Bm-CPI-2, a cystatin homolog secreted by the filarial parasite *Brugia malayi*, inhibits class II MHC-restricted antigen processing. *Curr Biol.* (2001) 11:447–51. 10.1016/S0960-9822(01)00118-X 11301256

[87] Sun Y Liu G Li Z Chen Y Liu Y Liu B Modulation of dendritic cell function and immune response by cysteine protease inhibitor from murine nematode parasite *Heligmosomoides polygyrus*. *Immunology.* (2013) 138:370–81. 10.1111/imm.12049 23240853 PMC3719947

[88] Schönemeyer A Lucius R Sonnenburg B Brattig N Sabat R Schilling K Modulation of human T cell responses and macrophage functions by onchocystatin, a secreted protein of the filarial nematode *Onchocerca volvulus*. *J Immunol.* (2001) 167:3207–15. 10.4049/jimmunol.167.6.3207 11544307

[89] Dainichi T Maekawa Y Ishii K Zhang T Nashed BF Sakai T Nippocystatin, a cysteine protease inhibitor from *Nippostrongylus brasiliensis*, inhibits antigen processing and modulates antigen-specific immune response. *Infect Immun.* (2001) 69:7380–6. 10.1128/IAI.69.12.7380-7386.2001 11705911 PMC98825

[90] Ziegler T Rausch S Steinfelder S Klotz C Hepworth MR Kühl AA Novel regulatory macrophage induced by a helminth molecule instructs IL-10 in CD4 ^+^ T cells and protects against mucosal inflammation. *J Immunol.* (2015) 194:1555–64. 10.4049/jimmunol.1401217 25589067

[91] Pfaff AW Schulz-Key H Soboslay PT Taylor DW MacLennan K Hoffmann WH. *Litomosoides sigmodontis* cystatin acts as an immunomodulator during experimental filariasisq. *Int J Parasitol.* (2002) 32:171–8. 11812494 10.1016/s0020-7519(01)00350-2

[92] Coronado S Zakzuk J Regino R Ahumada V Benedetti I Angelina A *Ascaris lumbricoides* cystatin prevents development of allergic airway inflammation in a mouse model. *Front Immunol.* (2019) 10:2280. 10.3389/fimmu.2019.02280 31611876 PMC6777510

[93] Schnoeller C Rausch S Pillai S Avagyan A Wittig BM Loddenkemper C Helminth immunomodulator reduces allergic and inflammatory responses by induction of IL-10-producing macrophages. *J Immunol.* (2008) 180:4265–72. 10.4049/jimmunol.180.6.4265 18322239

[94] Daniłowicz-Luebert E Steinfelder S Kühl AA Drozdenko G Lucius R Worm M A nematode immunomodulator suppresses grass pollen-specific allergic responses by controlling excessive Th2 inflammation. *Int J Parasitol.* (2013) 43:201–10. 10.1016/j.ijpara.2012.10.014 23174104

[95] Yang J Zhao J Yang Y Zhang L Yang X Zhu X *Schistosoma japonicum* egg antigens stimulate CD4 ^+^ CD25 ^+^ T cells and modulate airway inflammation in a murine model of asthma: *S. japonicum* eggs prevent asthma by Treg. *Immunology.* (2007) 120:8–18. 10.1111/j.1365-2567.2006.02472.x 17042799 PMC1890919

[96] Haeberlein S Obieglo K Ozir-Fazalalikhan A Chayé MAM Veninga H Vlugt LEPM Schistosome egg antigens, including the glycoprotein IPSE/alpha-1, trigger the development of regulatory B cells. *PLoS Pathog.* (2017) 13:e1006539. 10.1371/journal.ppat.1006539 28753651 PMC5550006

[97] Cardoso LS Oliveira SC Góes AM Oliveira RR Pacífico LG Marinho FV *Schistosoma mansoni* antigens modulate the allergic response in a murine model of ovalbumin-induced airway inflammation: *S. mansoni* antigens modulate allergy. *Clin Exp Immunol.* (2010) 160:266–74. 10.1111/j.1365-2249.2009.04084.x 20132231 PMC2857950

[98] Gomez-Escobar N Gregory WF Maizels RM. Identification of tgh-2, a filarial nematode homolog of *Caenorhabditis elegans* daf-7 and human transforming growth factor β, expressed in microfilarial and adult stages of Brugia malayi. *Infect Immun.* (2000) 68:6402–10. 10.1128/IAI.68.11.6402-6410.2000 11035752 PMC97726

[99] Zang X Taylor P Wang JM Meyer DJ Scott AL Walkinshaw MD Homologues of human macrophage migration inhibitory factor from a parasitic nematode: gene cloning, protein activity, and crystal structure. *J Biol Chem.* (2002) 277:44261–7. 10.1074/jbc.M204655200 12221083

[100] Tan THP Edgerton SAV Kumari R Mcalister MSB Rowe SM Nagl S Macrophage migration inhibitory factor of the parasitic nematode *Trichinella spiralis*. *Biochem J.* (2001) 357(Pt 2):373–83. 11439086 10.1042/0264-6021:3570373 PMC1221963

[101] Vermeire JJ Cho Y Lolis E Bucala R Cappello M. Orthologs of macrophage migration inhibitory factor from parasitic nematodes. *Trends Parasitol.* (2008) 24:355–63. 10.1016/j.pt.2008.04.007 18603473 PMC3615561

[102] Cho MK Park MK Kang SA Park SK Lyu JH Kim D-H TLR2-dependent amelioration of allergic airway inflammation by parasitic nematode type II MIF in mice. *Parasite Immunol.* (2015) 37:180–91. 10.1111/pim.12172 25559209

[103] Park SK Cho MK Park H-K Lee KH Lee SJ Choi SH Macrophage migration inhibitory factor homologs of *Anisakis simplex* suppress Th2 response in allergic airway inflammation model via CD4 ^+^ CD25 ^+^ Foxp3 ^+^ T cell recruitment. *J Immunol.* (2009) 182:6907–14. 10.4049/jimmunol.0803533 19454687

[104] Araújo CA Perini A Martins MA Macedo MS Macedo-Soares MF. PAS-1, a protein from *Ascaris suum*, modulates allergic inflammation via IL-10 and IFN-γ, but not IL-12. *Cytokine.* (2008) 44:335–41. 10.1016/j.cyto.2008.09.005 19008120

[105] Itami DM Oshiro TM Araujo CA Perini A Martins MA Macedo MS Modulation of murine experimental asthma by *Ascaris suum* components. *Clin Exp Allergy.* (2005) 35:873–9. 10.1111/j.1365-2222.2005.02268.x 16008672

[106] De Araújo CAA Perini A Martins MA Macedo MS Macedo-Soares MF. PAS-1, an *Ascaris suum* protein, modulates allergic airway inflammation via CD8+ γδTCR+ and CD4+ CD25+ FoxP3+ T Cells: PAS-1 suppresses allergic responses via TREG cells. *Scand J Immunol.* (2010) 72:491–503. 10.1111/j.1365-3083.2010.02465.x 21044123

[107] Holgate ST Polosa R. Treatment strategies for allergy and asthma. *Nat Rev Immunol.* (2008) 8:218–30. 10.1038/nri2262 18274559

[108] Barrett NA Rahman OM Fernandez JM Parsons MW Xing W Austen KF Dectin-2 mediates Th2 immunity through the generation of cysteinyl leukotrienes. *J Exp Med.* (2011) 208:593–604. 10.1084/jem.20100793 21357742 PMC3058587

[109] Laan LC Williams AR Stavenhagen K Giera M Kooij G Vlasakov I The whipworm (*Trichuris suis*) secretes prostaglandin E2 to suppress proinflammatory properties in human dendritic cells. *FASEB J.* (2017) 31:719–31. 10.1096/fj.201600841R 27806992 PMC5240662

[110] Alcantara-Neves NM Veiga RV Dattoli VCC Fiaccone RL Esquivel R Cruz ÁA The effect of single and multiple infections on atopy and wheezing in children. *J Allergy Clin Immunol.* (2012) 129:359–67.e3. 10.1016/j.jaci.2011.09.015 22035877 PMC5015705

[111] Cooper PJ Chico ME Vaca MG Moncayo A-L Bland JM Mafla E Effect of albendazole treatments on the prevalence of atopy in children living in communities endemic for geohelminth parasites: a cluster-randomised trial. *Lancet.* (2006) 367:1598–603. 10.1016/S0140-6736(06)68697-2 16698413

[112] Evans H Mitre E. Worms as therapeutic agents for allergy and asthma: understanding why benefits in animal studies have not translated into clinical success. *J Allergy Clin Immunol.* (2015) 135:343–53. 10.1016/j.jaci.2014.07.007 25174866

